# Integrating health belief model and theory of planned behavior to assess COVID-19 vaccine acceptance among urban slum people in Bangladesh

**DOI:** 10.1371/journal.pone.0290412

**Published:** 2023-12-20

**Authors:** Muhammad Mainuddin Patwary, Asma Safia Disha, Mahadi Hasan, Mondira Bardhan, Mehedi Hasan, Faiza Imam Tuhi, Sama Jamila Rahim, Md. Navid Newaz, Sardar Al Imran, Md. Zahidul Haque, Md. Riad Hossain, Md Pervez Kabir, Sarya Swed

**Affiliations:** 1 Environment and Sustainability Research Initiative, Khulna, Bangladesh; 2 Environmental Science Discipline, Khulna University, Khulna, Bangladesh; 3 Department of Environmental Science and Management, North South University, Bashundhara, Dhaka, Bangladesh; 4 Department of Environmental Science, Bangladesh Agricultural University, Mymensingh, Bangladesh; 5 Department of Statistics, University of Dhaka, Dhaka, Bangladesh; 6 Development Studies Discipline, Khulna University, Khulna, Bangladesh; 7 Institute of Disaster Management, Khulna University of Engineering & Technology, Khulna, Bangladesh; 8 Faculty of Medicine, Aleppo University, Aleppo, Syria; Sunway University, MALAYSIA

## Abstract

**Introduction:**

The vaccination against coronavirus disease 2019 (COVID-19) has been identified as a promising strategy to reduce the severity of the pandemic. Despite the safe and effective COVID-19 vaccines, bringing socioeconomically disadvantaged people under vaccination coverage has been challenging for developing countries like Bangladesh. Therefore, this study explored the determinants of vaccine acceptance among urban slum residents of Bangladesh using the Health Belief Model (HBM) and Theory of Planned Behavior (TPB).

**Methods:**

A face-to-face survey of 400 urban slum dwellers in two large cities in Bangladesh was conducted between July 5 to August 5, 2021. The questionnaire included vaccine acceptance, socio-demographics, health-related characteristics, trust in health authorities, reasons for vaccine hesitancy, and dimensions of HBM and TPB frameworks. Hierarchical logistic regression was performed to evaluate the association between these characteristics and vaccination acceptance.

**Results:**

Around 82% (n = 327) of respondents were willing to accept the COVID-19 vaccine. In a fully adjusted model, respondents with secondary level education had higher intention (OR = 46.93, 95%CI = 1.21–1807.90, p < 0. 05) to accept COVID-19 vaccine. Respondents with bad (OR = 0.11, 95%CI = 0.01–0.35, p<0.05) or very bad (OR = 0.01, 95%CI = 0.01–0.35, p<0.05) health conditions were less interested in the COVID-19 vaccination. In regard to HBM dimensions, greater perceived susceptibility (OR = 1.75, 95% CI = 1.12–2.75, p < 0.05), and perceived benefits (OR = 3.28, 95% CI = 1.17–6.00, p < 0.001) were associated with a greater willingness to get vaccinated. In regard to TPB, higher self-efficacy in preventing illness without the vaccine increased the desire to get vaccinated (OR = 1.55, 95% CI = 1.02–2.37, p < 0.05). Fear of unknown side effects, religious beliefs, contraindications to vaccination, and insufficient information on the vaccine were the main reasons for vaccine hesitancy.

**Conclusions:**

These findings offer valuable insights for policymakers in Bangladesh to design targeted interventions that address vaccine hesitancy and increase vaccination acceptability among socially disadvantaged individuals in urban areas. Strategies should focus on providing accurate and accessible information about the vaccine, communicating its positive impact effectively, engaging with religious leaders to address misconceptions, and tailoring vaccination campaigns to meet the unique needs of different demographic groups.

## 1. Introduction

The novel coronavirus disease (COVID-19) pandemic, declared by the World Health Organization (WHO) as a global emergency, has affected 228 countries with more than 178 million confirmed cases and nearly 3.90 million deaths reported as of June 20, 2021 [1]. Given its highly infectious nature, COVID-19 has brought unprecedented public health risks as well as a socioeconomic catastrophe to all nations [2]. Bangladesh has also been impacted, with a significant number of cases and deaths [1]. Although Governments have implemented several preventive measures [3, 4], vaccination remains the key strategy to achieve herd immunity and control the spread of the COVID-19 infections [5].

Global vaccination coverage of around 70% is crucial to control the spread of COVID-19 pandemic [6]. As of June 2021, 2.69 billion vaccine doses have been distributed worldwide [7]. However, vaccination uptake is still inadequate [8, 9]. Further, low-income countries face vaccination disparities, as merely 25.9% of individuals in these countries have received at least one dose, due to supply chain dynamics, limited accessibility, and misconceptions about the vaccine [10]. Although Bangladesh has a history of successful immunization campaigns [11], it faced challenges in the initial stages of the COVID-19 vaccination drive. However, the government procured vaccines from multiple sources and has made significant efforts to vaccinate the population. As of December 2021, about 50% of the population received one dose and 25% received two doses [12].

Vaccine hesitancy, defined as a delay in acceptance or refusal of vaccination despite its availability, is a global health concern that has been observed even prior to the COVID-19 pandemic [13, 14]. Understanding the factors influencing vaccine acceptance is crucial, especially considering the documented low vaccination rates in high income countries such as Kuwait (23.6%), Jordan (28.4%), Italy (53.7%), Russia (54.9%), Poland (56.3%), and France (58.9%) (19). However, studies on vaccine hesitancy are limited in low-and-middle-income countries.

Earlier studies in Bangladesh have reported varying COVID-19 vaccine acceptance rates. One study among the general population found an acceptance rate of 85% [15], while another study among healthcare professionals reported a rate of less than 50% of vaccine acceptance [16]. The difference in acceptance rates between these studies could be attributed to factors such as the timing of the studies. The study among the general population [15] took place after the vaccine program had started, which may have influenced people’s perception of the vaccine’s benefits. On the other hand, the study among healthcare workers was conducted before the vaccination program began, potentially leading to vaccine hesitancy due to concerns about safety and efficacy [16]. Importantly, the study revealed that 43.8% respondents preferred to wait and observe vaccine effects on others before receiving it themselves [16]. Given the vulnerable healthcare system in Bangladesh [2] and the World Health Organization’s strategy to vaccinate 70% of the global population by mid-2022 [17], it is crucial to identify vaccine reluctance rates and factors associated with it to ensure successful mass vaccination efforts.

**Fig 1:**
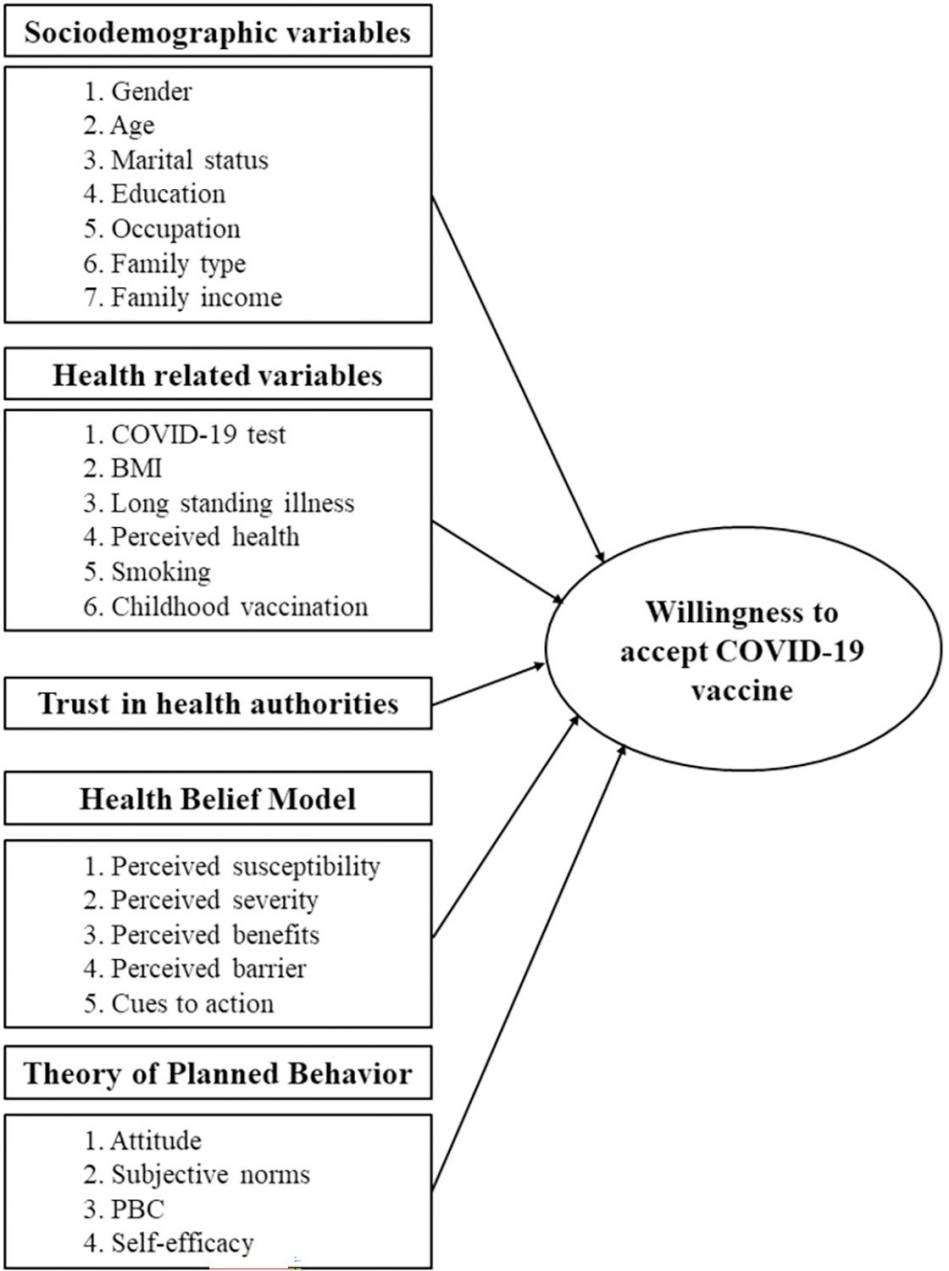
A conceptual framework for the determinants of COVID-19 vaccine acceptance among urban slum dwellers.

The Health Belief Model (HBM) and the Theory of Planned Behavior (TPB) are essential tools for predicting vaccination decisions. The HBM is a widely used framework for studying the nexus between individual health behavior and the acquisition of health care services [18, 19]. Several earlier studies reported the use of the HBM model to predict vaccination behavior, in particular, Ebola [20], H1N1 [21], and COVID-19 [22]. The Health Belief Model (HBM) suggests that people’s decision to engage in health behavior, such as getting vaccinated is determined by the individual’s perception of getting infected by the virus (e.g., COVID-19 in the absence of vaccine (“perceived susceptibility”), beliefs on developing serious health difficulties if infected (“perceived severity”), beliefs on vaccine would be effective against the virus (e.g., COVID-19) (“perceived benefits”), beliefs on vaccine would not cause any side effects (“perceived barriers”), along with the perceptions that vaccination recommended by healthcare professionals (“cues to action”) [19]. The Theory of Planned Behavior (TPB) states that an individual’s behavior is primarily influenced by their belief system [23]. In the context of COVID-19, the TPB model suggests that the intention to get vaccinated depends on favorable attitudes about vaccination (“attitudes”), presence of key persons who support vaccination (“subjective norms”), perceptions of the extent to which vaccination is under control (“perceived behavior control”), and beliefs on avoiding illness by practicing regularly preventive health measures (“self-efficacy”) [24]. Further, vaccine hesitancy may be explained by the public trust in government health officials. Trust in healthcare authorities and government institutions plays a crucial role in shaping individuals’ attitudes and behaviors towards vaccines [25]. Lazarus et al. [26] found strong evidence of trust in government having a significant impact on vaccine hesitancy in a study across 19 countries [26]. Similarly, Lindholt et al. [27] conducted an 8-country study and observed that trust in government further contributed to vaccine hesitancy [27]. A conceptual diagram for the determinants of COVID-19 vaccine acceptance using HBM and TPB framework for this study presented in Fig 1.

Approximately 1 billion people worldwide live in slums [28], characterized by overcrowded living conditions and limited access to basic amenities such as sanitation, water, and healthcare facilities [29]. Slum populations have been disproportionately affected by the COVID-19 pandemic [30], with high population density and inadequate sanitation contributing to increased transmission risk [31]. Studies in India have shown that over 50% of individuals in these communities have been exposed to the virus [32, 33], while in Brazil, slum residents accounted for a significant proportion of COVID-19 deaths despite representing a smaller portion of the city’s population [30]. Additionally, the economic consequences of the pandemic, including rising unemployment rates, further compound the challenges faced by slum communities in adhering to preventive measures [34]. Further, these communities faced low-vaccination rate due to lack of access to vaccination centers, long queues, and inadequate vaccine supplies [35]. Consequently, it is imperative to investigate the understanding of vaccine acceptance among slum populations and the barriers they encounter.

In Bangladesh, 47% of population resides in informal settlements, highlighting the significant presence of urban slums [36]. These communities have long faced financial challenges and limited access to healthcare services due to inequitable conditions and the overall economy. Disturbingly, data indicates that 75% of the 2.2 million slum residents in the country live in a single room, with 45% experiencing regular infectious and parasitic diseases [37]. On top of that, the COVID-19 pandemic has further exacerbated their hardships [38]. Complicating matters, slum-dwellers face uncertainties, lack of reliable information, rumors, and misconceptions about COVID-19 treatment and vaccines, hindering their willingness to seek healthcare services, including vaccination [39, 40]. In this situation, the potential unwillingness of the slum-dwellers to be vaccinated against COVID-19 might leave them more vulnerable to the virus. Given the situation, the potential vaccine hesitancy among slum residents increases their vulnerability to COVID-19, highlighting the importance of prioritizing their vaccination to effectively combat the pandemic and address any concerns related to vaccine hesitancy, ensuring their awareness and engagement.

Few studies in Bangladesh focused on the vaccine acceptance rate, including the general population [15, 41, 42] and healthcare professionals [16]. However, each of these studies investigated the vaccination rates of socioeconomically privileged people of Bangladesh. On the other hand, a study reported the vaccination status of the low-income population in Bangladesh [43]. However, their study participants were from a mixed population, including metropolitan cities, rural, semi-urban, and slum areas. Thus, it was not identified in the article how many respondents they collected from slums only. Consequently, they did not determine separately what socioeconomic and behavioral factors influenced the vaccination decision among the slum dwellers. However, it is necessary to evaluate the extent and influencing factors of the COVID-19 vaccination scenario among the slum people of urban areas. Thus, the objective of this study was to assess the vaccine acceptance rates among slum dwellers in urban areas of Bangladesh and identify the behavioral factors that influence their vaccination decision, using the integrated Health Belief Model (HBM) and the Theory of Planned Behavior (TPB) theoretical model.

## 2. Methods

### 2.1. Study design and participants

A cross-sectional study was conducted when the country experienced a devastating second wave of the pandemic. A face-to-face survey with maintaining proper social distancing was conducted between July 5 and August 5, 2021, before starting the vaccination in urban slums. The inclusion criteria for being eligible for this study were included as participants must be urban slum dwellers in Bangladesh, being at least 18 years old and must not have received the first dose of a COVID-19 vaccine. Exclusion criteria were participants who do not meet the criteria of being urban slum dwellers in Bangladesh and below 18 years of age. The data were collected from the urban slums of Dhaka and Khulna City of Bangladesh. The data collection process involved a team of six trained interviewers who were carefully selected based on their experience in conducting surveys and their familiarity with the local context. Prior to the interviews, all interviewers underwent a comprehensive training program that covered the objectives of the study, interview techniques, ethical considerations, and data collection protocols. The training aimed to ensure consistency and standardization in the data collection process.

To ensure a representative sample from the slum population, we employed a multistage random sampling technique. Firstly, we obtained a comprehensive list of slum areas in the urban region, including Dhaka and Khulna City, from the slum census of Bangladesh Bureau of Statistics [44]. Based on accessibility considerations, we selected a predetermined number of slum areas as primary sampling units (PSUs). Specifically, we chose five slum areas (Santibagh, Malibagh, Anis, Khilgaon, Maya kanon, and Abu Slum) from Dhaka City and three slum areas (Sonargaon, Boyra, and Railway Slum) from Khulna City. Since we could not conduct any pilot study before to collect household information separately, we conducted a thorough consultation with community leaders and community organizers of the each selected slums. By consulting with these key stakeholders, we obtained valuable insights into the overall slum population settings. Based on this information, we selected households that were eligible for participation in our study. Finally, we proceeded to randomly select eligible participants for the structured interviews from the selected households.

In the absence of prior research employing our set of measures, we employed an internet calculator to determine our sample size [45]. We used the recommended degree of caution (50 percent) for the proportion of our sample that exhibited our variable of interest. Therefore, using an online sample size calculator (https://statulator.com/ accessed on July 1, 2021), we determined the minimum required number of respondents to be 385, based on a 10% non-response rate, 5% precision, and 50% proportion, with a confidence interval of 95% for the total population size of 2.2 million urban slum population in Bangladesh [44].

Therefore, a total of 410 responses were collected, of which 400 respondents were used for the final analysis since the remaining ten respondents were failed to meet the inclusion criteria. Prior to completing the survey, all participants provided their written consent. Participants could opt out form the survey at any time without any consent. In addition, this survey did not request any personal information from respondents, ensuring they could not be identified. This study was approved by the research ethics committee of the Institute of Disaster Management, Khulna University of Engineering & Technology, Khulna, Bangladesh.

### 2.2. Measures

In this study, a structured questionnaire was used to collect data on various factors related to socioeconomic variables, health-related factors, COVID-19 vaccine acceptance intention, health belief measures, trust in health authorities, and reasons for COVID-19 vaccination reluctance. The questionnaire consisted of a total of 46 questions administered to the respondents. To ensure inclusivity and ease of response, the questionnaire was translated into the local language (Bengali), to facilitate data collection. This step aimed to enhance participants’ understanding and engagement with the questionnaire, considering the cultural and linguistic context of the study population.

The sociodemographic variables included in the questionnaire were gender, age, marital status, education, occupation, family type, and monthly family income. In addition to sociodemographic variables, the questionnaire also included several health-related variables. These variables encompassed COVID-19 test positivity, body mass index (BMI), presence of long-term illness(es), subjective health status, smoking habit, and childhood vaccination status. The Health Belief Model [43, 44] and Theory of Planned Behavior [27, 45, 46] were considered health belief measures for this study (Fig 1).

### 2.3. Willingness to accept vaccine and reasons not to receive the vaccine

We used a single item to assess the participant’s willingness to receive a COVID-19 vaccine by asking, "Will you take the Covid-19 vaccine when it becomes available?". The possible responses were ’Yes,’ ’No,’ or ’Not sure’ [46, 47]. The participant’s choice was grouped into three groups: (1) willing to take the vaccination (answer = ’Yes’), (2) hesitant about vaccine acceptance (response = ’Not sure’), and (3) unwilling to receive the vaccine (response = ’No’). We grouped the latter two choices (’Uncertain’ and ’No’) and deemed them vaccination reluctance. Therefore, respondents were asked why they would reject or hesitate to get the vaccination. The possible reasons were " I do not need the vaccine because I am healthy and low risk for infection, "natural immunity lasts longer than a vaccination," "I prefer other people get the vaccine first." and "I doubt the vaccine’s effectiveness," "I fear the unknown effects of vaccines in the future," "I have contraindications to the vaccine (e.g., allergies, high blood pressure, diabetes," "I have insufficient information regarding the vaccine," and "the financial cost is a hindrance if the vaccine is not free." Respondents had the provision to identify one or more of these possible reasons.

### 2.4. Trust in health authorities

Two items were used to measure the public’s trust in the country’s health authorities to control COVID-19 transmission (Cronbach α = 0.87). These were "I trust in the country’s health authorities" and "I trust in healthcare staff for COVID-19 control". All questions were graded on a 5-point scale ranging from strongly disagree (1) to strongly agree (5). The total score was determined by averaging the two ratings, with higher ratings reflecting more satisfaction in the country’s health officials.

### 2.5. Health Belief Model (HBM) variables

Five dimensions of the Health Belief Model (HBM), including perceived susceptibility, perceived severity, perceived benefits, perceived barriers, and cues to action, were used in this study [48, 49]. The items for HBM model for this study were adapted from previous published literatures [24]. Two items were used to measure the perceived susceptibility. Items were "I may get infected if I do not COVID-19 vaccinated" and "My family may get infected if they do not COVID-19 vaccinated". The internal consistency of this measure was satisfactory (Cronbach α = 0.83). Perceived severity was measured using two items with a Cronbach α = 0.67. Items included "Complications from COVID-19 are very serious" and "Recovering from COVID-19 would take a long time". Two items were included to measure perceived benefit with an acceptable internal consistency score (Cronbach α = 0.83). Items were "The vaccine will reduce my fear of contagion" and "The vaccine will be highly effective to prevent the spread of COVID-19." Perceived barriers included two items: "COVID-19 vaccine would have possible side effects" and "I am doubtful about the efficacy of the COVID-19 vaccine" with a good internal consistency score (Cronbach α = 0.71). Cues to action were measured using four items, including ("I would take the vaccine if recommended by a Doctor," "I would take the vaccine if recommended by a Public figure/political leader," and "I would take the vaccine if Ministry of Health Publish any guideline, " and " I would take the vaccine if recommended by family/friends") with a good internal consistency value (Cronbach α = 0.87).

Health Belief Model dimensions were rated on a five-point scale from "1" (strongly disagree) to "5" (strongly agree). The item scores were averaged to get an overall score for each dimension, with higher values indicating higher levels of that dimension. The perceived barrier scale, however, was coded reverse direction, such that greater scores indicated lower levels of perceived barrier among the participants [24].

### 2.6. Theory of Planned Behavior (TPB) variables

While the original Theory of Planned Behavior (TPB) focused on three factors—attitudes, subjective norms, and perceived behavior control (PBC), recent studies have expanded its scope to include a fourth factor—self-efficacy [48, 50]. Two items were used to measure the attitudes following the research by [24]: "Getting vaccinated is a tedious and time-consuming process" and "I think COVID-19 vaccine probably will not work" with a Cronbach α = 0.47. A single item was used to assess subjective norms: "My family & friends will respond positively to vaccination." Following previous research [24, 51], perceived behavior control (PBC) was measured by asking the respondent about the "Decision on taking vaccination is entirely up to me." The self-efficacy was also measured using a single item adapted from past research [24, 52] and included "If I take all the necessary precautions (disinfection of hands, etc.), I do not need to be vaccinated" for self-efficacy. Each TPB item was graded on a 5-point scale ranging from"1" (strongly disagree) to"5" (strongly agree). The response to each item was averaged to determine the total score for each TPB dimension, except for self-efficacy, which was reverse coded.

### 2.7. Covariates

Gender, age, marital status, education, occupation, family type and monthly family income were included as sociodemographic variables. Gender was determined by inquiring if the participant was male or female. Marital status was classified as single, married or divorced. Age was measured as a continuous variable. Educational level of the respondents was categorized into four classes: (1) no formal education (2) currently primary level, (3) secondary school certificate (SSC) level, or (4) college or higher degree. Family type was classified into a nuclear or joint family. Respondents were asked to indicate their occupation as currently unemployed, student or worker, day laborer, small business or housewife. Monthly family income was categorized as ≤5000 BDT, 5001–10000 BDT, 10001–15000 BDT and, >15000 BDT.

The COVID-19 test positivity, body mass index (BMI), long-term illness(es), subjective health status, smoking habit, and childhood vaccination status were considered as health-related variables in this study. Respondents were asked to identify whether they were susceptible to COVID-19, if they had a chronic condition, whether they smoked, and if they had been immunized as children. Body Mass Index (BMI) was determined using height (m^2^) and weight (kg) of the respondents. The perceived health status of the respondent was rated as very good, good, fair, or poor.

### 2.8. Data analysis

Descriptive analyses were performed for various sociodemographic variables and also by the vaccine acceptance categories. To identify the statistically significant independent variables for subsequent analysis, a t-test or chi-square test, or Kruskal-Wallis test, was performed depending on the nature of the variables. The factors associated with vaccination decision was determined using a hierarchical logistic regression model after adjusting for other potential variables. In this study, the dependent variable was the willingness to accept the vaccine (willingness to accept vaccine = 1 and unwillingness or undecided to vaccinate = 0). On the other hand, the independent variables were sociodemographic, health, public trust, and HBM and TPB dimensions. Each variable with a p<0.05 in univariate analysis was included in the final analyses. These independent variables were clustered into five blocks using a forward stepwise regression. Blocks 1 & 2 included the sociodemographic and health-related variables, respectively. Indicators for public trust in health authorities were placed in block 3. Block 4 & 5 considered the HMB and TPB dimensions, respectively. Variables were included in the model based on our research objectives and theoretical framework. We included the HBM variables first to assess their individual contributions in explaining vaccine acceptance, considering sociodemographic, health and trust related factors. We aimed to determine if the HBM variables could explain additional variance in vaccine acceptance beyond sociodemographic factors. The TPB variables were subsequently added to the model to investigate their incremental contribution in explaining vaccine acceptance, after accounting for sociodemographic, health, trust related factors and the HBM variables. A two-tailed test with a p-value of less than 0.05 was deemed statistically significant. The reasons for vaccine hesitancy were explained as frequencies of those reported undecided or unwilling to accept vaccine. The IBM SPSS software version 26 was used to conduct all analyses.

## 3. Results

### 3.1. Sociodemographic features and acceptance of the vaccine

Table 1 summarizes the demographics and health status of the participants based on vaccination decisions in this study. Among the participants, more than half (56.8%) of the participants were female, relatively young (mean age: 33.43±11.25) (range 18–77 years old) and married (90.0%). More than half had no formal education (52.5%), and few attended college (6.3%). Around 29% of the surveyed people worked as day laborers, whereas 11% were jobless. Only 3.5% of our surveyed population were students. There were 91.8% of the nuclear family types, with monthly salaries ranging from 5001 to 10000 BDT(46–92 USD) (70.9%). Only 7.8% were previously infected by COVID-19. The average Body Mass Index (BMI) of all participants was 22.50±3.61. Nearly two-thirds of the respondents reported having a long-term sickness, while the vast majority confirmed that their health was in the "Fair to Very Good" condition (21.5 to 33.3%). Most of the respondents (68.8%) did not smoke, and nearly all of the respondents had received their childhood vaccination (81.8%).

**Table 1:** Descriptive statistics of respondents by the willingness to accept the COVID-19 vaccine (N = 400).

Variables	Total N = 400 (%)	Vaccine acceptance		χ^2^ ^a^	p-value
		Intended n = 327 (%)	Undecided or unwilling n = 73 (%)		
**Sociodemographic characteristics**					
**Gender**				7.631	0.006**
Male	173 (43.2)	152 (46.5)	21 (28.8)		
Female	227 (56.8)	175 (53.5)	52 (71.2)		
**Age**	33.43 (±11.25)	31.97 (±10.07)	39.93 (±13.77)	63.090	0.009**
**Marital status**				2.411	0.300
Single	35 (8.8)	31 (9.5)	4 (5.5.)		
Married	360 (90.0)	291 (89.0)	69 (94.5)		
Divorced	5 (1.2)	5 (1.5)	0		
**Education**				17.828	0.000***
No formal education	210 (52.5)	157 (48.0)	53 (72.6)		
Primary level	115 (28.7)	99 (30.3)	16 (21.9)		
Secondary School Level	50 (12.5)	49 (15.0)	1 (1.4)		
≥ College	25 (6.3)	22 (6.7)	3 (4.1)		
**Occupation**				18.563	0.002**
Unemployed	44 (11.0)	27 (8.2)	17 (23.3)		
Student	14 (3.5)	11 (3.4)	3 (4.1)		
Worker	106 (26.5)	90 (27.5)	16 (21.9)		
Day labor	116 (29.0)	98 (30.0)	18 (24.7)		
Small business	31 (7.8)	30 (9.2)	1 (1.4)		
Housewife	89 (22.2)	71 (21.7)	18 (24.7)		
**Family type**				0.212	0.646
Nuclear	367 (91.8)	301 (92.0)	66 (90.4)		
Joint	33 (8.2)	26 (8.0)	7 (9.6)		
**Monthly family income (BDT)**				7.064	0.070
≤5000 BDT (≤45 USD)	90 (22.5)	68 (20.8)	22 (30.1)		
5001–10000 BDT (46–92 USD)	174 (43.5)	139 (42.5)	35 (47.9)		
10001–15000 BDT (93–138 USD)	89 (22.2)	77 (23.5)	12 (16.4)		
>15000 BDT (>138 USD)	47 (11.8)	43 (13.2)	4 (5.5)		
**Health-related variables**					
**COVID-19 test positivity**				0.644	0.422
No	369 (92.2)	300 (91.7)	69 (94.5)		
Yes	31 (7.8)	27 (8.3)	4 (5.5)		
**BMI**	22.50 (±3.61)	22.45 (±3.69)	22.71 (±3.24)	189.033	0.734
**Long-standing illness(es)**				14.100	0.000***
No	257 (64.2)	224 (68.5)	33 (45.2)		
Yes	143 (35.8)	103 (31.5)	40 (54.8)		
**Perceived health condition**				59.787	0.000***
Very good	133 (33.3)	118 (36.1)	15 (20.5)		
Good	138 (34.5)	121 (37.0)	17 (23.3)		
Fair	86 (21.5)	70 (21.4)	16 (21.9)		
Bad	26 (6.5)	14 (4.3)	12 (16.4)		
Very bad	17 (4.2)	4 (1.2)	13 (17.8)		
**Smoking**				5.9321	0.052
No smoking	275 (68.8)	219 (67.0)	56 (76.7)		
Current smoker	108 (27.0)	96 (29.4)	12 (16.4)		
Former smoker	17 (4.2)	12 (3.7)	5 (6.8)		
**Childhood vaccination(s)**				15.316	0.000***
No	73 (18.2)	48 (14.7)	25 (34.2)		
Yes	327 (81.8)	279 (85.3)	48 (65.8)		

BDT, Bangladeshi Taka (currency); USD, United States Dollar (currency); BMI, Body Mass Index

Data are presented as N (%) or mean (±SD)

^a^ Chi-square test

**p<0.01

***p<0.001

Around 81.75% (n = 327) of respondents planned to have COVID-19 vaccinated. Seven variables exhibited disparities across vaccination acceptance groups during the analysis of demographic and health-related variables. There was a higher acceptance rate among females (χ^2^ = 7.631, p < 0.001), young individuals (χ^2^ = 63.090, p < 0.001), respondents who had no formal education (χ^2^ = 17.828, p < 0.001), who had worked as day laborers in the past (χ^2^ = 18.563, p < 0.05), who had not suffered from any long-term diseases (χ^2^ = 14.100, p < 0.001), people who perceived "Good" health condition (χ^2^ = 59.787, p < 0.05), and those who had received childhood vaccination (χ^2^ = 15.316, p < 0.001).

### 3.2. Univariate analyses of HBM and TPB dimensions

Univariate results of public trust in health authorities, HBM, and TPB regarding COVID-19 vaccine acceptance are summarized in Table 2. Public trust in health authorities and TPB domains, except PBC differed significantly between the two groups (p > 0.05). Substantial variations were also seen across groups in every aspect of the HBM. Those who planned to be vaccinated were more sensitive to the virus (χ^2^ = 139.079, p < 0.001), perceived it to be more severe (χ^2^ = 11.308, p < 0.05), felt vaccination to be more helpful (χ^2^ = 139.065, p < 0.001), and had more cues to action (χ^2^ = 74.252, p < 0.001). In contrast, individuals who were advised to be vaccinated reported fewer perceived barriers (χ^2^ = 55.972, p < 0.001). Besides, all TPB measures except for PBC (p > 0.05) showed significant differences between the groups. We found that those who planned to be vaccinated reported higher subjective norms (χ^2^ = 45.714, p < 0.001). Individuals with more negative attitudes (χ^2^ = 42.911, p < 0.001) towards COVID-19 were less likely to be vaccinated. Moreover, people with lower self-efficacy in their abilities to avoid illness showed lower vaccination intentions (χ^2^ = 33.198, p < 0.001).

**Table 2:** Univariate analysis of the dimensions of health behavior model (HBM) and theory of planned behavior (TPB), and COVID-19 vaccine acceptance.

Variables	Total (N = 400)	Vaccine acceptance		χ^2^ ^a^	p-value
		Intended (n = 327)	Undecided or Unwillingness (n = 73)		
	Mean (SD)	Mean (SD)	Mean (SD)		
**Trust in health authorities**	4.04 (0.89)	3.99 (0.87)	3.61 (0.96)	13.119	0.011**
**HBM**					
Perceived susceptibility	3.66 (1.21)	3.86 (0.95)	2.21 (1.15)	139.079	0.000***
Perceived severity	4.16 (0.76)	4.05 (0.73)	3.77 (0.89)	11.308	0.02*
Perceived benefits	3.85 (1.04)	3.93 (0.80)	2.50 (1.02)	139.065	0.000***
Perceived barriers (reverse coded)	2.34 (0.84)	2.68 (1.18)	1.66 (0.74)	55.972	0.000***
Cues to action	3.65 (0.99)	3.74 (0.85)	2.68 (1.07)	74.252	0.000***
**TPB**					
Attitude	3.51 (0.97)	3.14 (0.88)	3.77 (0.99)	42.911	0.000***
Subjective norms	3.92 (0.86)	4.02 (0.78)	3.49 (1.08)	45.714	0.000***
PBC	4.02 (1.13)	3.95 (1.17)	4.34 (0.92)	8.765	0.067
Self-efficacy (reverse coded)	3.35 (1.24)	3.52 (1.18)	2.63 (1.23)	33.198	0.000***

Data are presented as mean (±SD)

^a^ Chi-Square test

*p<0.05

**p<0.01

***p<0.001

### 3.3. Multivariate analysis of factors associated with COVID-19 vaccine acceptance

Hierarchical logistic regression findings are shown in Table 3. Sociodemographic, health related factors and trust to health authorities were included in the model as control variables. The first model explained 26% of the variation using sociodemographic information. The second model contributed 9% to analysis variation with sociodemographic and health related variables. The third model contributed only 1% of the variance by including trust in health authorities. The variation was increased by 30% in the fourth model when the HBM variables were included. In this model, secondary level education (OR = 45.15, 95%CI = 1.81–1125.32, p < 0.05), perceived susceptibility (OR = 1.8, 95% CI = 1.21–2.82, p<0.01), perceived benefits (OR = 3.06, 95% CI = 1.8–5.20, p<0.001), and cues to action (OR = 1.72, 95% CI = 1.09–2.70, p < 0.01) were associated with vaccine acceptance. However, respondents with perceived bad health condition (OR = 0.02, 95%CI = 0.01–0.52, p < 0.05) show reluctance to accept vaccine. The fifth and final model added TPB dimensions to see combined effects with HBM, which increased the explained variance by 2%. In combined model, respondents with secondary level education had higher intention (OR = 46.93, 95%CI = 1.21–1807.90, p < 0. 05) to accept COVID-19 vaccine than lower or no educated individuals. Respondents with bad (OR = 0.11, 95%CI = 0.01–0.35, p<0.05) or very bad (OR = 0.01, 95%CI = 0.01–0.35, p<0.05) health conditions were less interested in the COVID-19 vaccination. In regard to HBM dimensions, greater perceived susceptibility (OR = 1.75, 95% CI = 1.12–2.75, p < 0.05), and perceived benefits (OR = 3.28, 95% CI = 1.17–6.00, p < 0.001) were all associated with a greater willingness to get vaccinated. For TPB, only one component was shown to have a statistically significant relationship with vaccination acceptance. An increase in self-efficacy (If I take all the necessary precautions, I do not need to be vaccinated) in preventing illness without the vaccine increased the desire to get vaccinated (OR = 1.55, 95% CI = 1.02–2.37, p < 0.05).

**Table 3:** Hierarchical logistic regression analysis for determining the influencing factors of COVID-19 vaccine acceptance (N = 400).

Predictors	Model 1	Model 2	Model 3	Model 4	Model 5
	OR (95% CI)	OR (95% CI)	OR (95% CI)	OR (95% CI)	OR (95% CI)
**Sociodemographic factors**					
**Gender**					
Male	Ref.	Ref.	Ref.	Ref.	Ref.
Female	2.19* (1.06–4.54)	3.67* (1.10–12.25)	3.63* (1.09–12.10)	1.63 (0.34–7.67)	1.35 (0.26–6.84)
**Age**	0.94*** (0.91–0.97)	0.96* (0.92–0.99)	0.96* (0.92-.99)	0.98 (0.94–1.03)	0.98 (0.94–1.03)
**Education**					
No formal education	Ref.	Ref.	Ref.	Ref.	Ref.
Primary level	1.15 (0.56–2.35)	1.19 (0.54–2.62)	1.21 (0.54–2.72)	1.26 (0.44–3.54)	1.06 (0.36–3.14)
Secondary School Level	14.04* (1.38–142.62)	23.37* (1.77–307.93)	22.35* (1.79–277.91)	45.15* (1.81–1125.32)	46.93* (1.21–1807.90)
≥ College	5.73 (0.38–84.76)	6.14 (0.27–135.64)	5.71 (0.25–129.73)	5.48 (0.02–120.16)	5.73 (0.05–6348.13)
**Occupation**					
Unemployed	Ref.	Ref.	Ref.	Ref.	Ref.
Student	0.12 (0.01–2.38)	0.06 (0.01–1.88)	0.06 (0.00–1.82)	0.03 (0.00–6.91)	0.04 (0.00–35.22)
Worker	2.68* (1.01–7.07)	1.90 (0.64–5.62)	2.04 (0.68–6.08)	2.15 (0.47–9.86)	2.90 (0.62–13.56)
Day labor	2.79* (1.08–7.24)	2.14 (0.73–6.25)	2.18 (0.74–6.42)	10.37 (0.46–230.51)	2.29 (0.44–11.98)
Small business	9.38* (1.00–87.44)	5.85 (0.59–57.58)	4.92 (0.49–48.83)	2.19 (0.54–10.62)	5.22 (0.28–95.00)
Housewife	1.87 (0.68–5.09)	1.50 (0.48–4.69)	1.37 (0.43–4.31)	2.02 (0.44–9.20)	2.18 (0.40–11.67)
**Health-related variables**					
**Long-standing illness(es)**					
No		Ref.	Ref.	Ref.	Ref.
Yes		0.85 (0.31–2.34)	0.97 (0.40–2.35)	1.72 (0.52–5.70)	2.37 (0.67–8.29)
**Perceived health condition**					
Very good		Ref.	Ref.	Ref.	Ref.
Good		1.23 (0.51–2.93)	1.36 (0.56–3.28)	0.86 (0.26–2.84)	0.83 (0.24–2.82)
Fair		0.85 (0.31–2.34)	.97 (0.34–2.73)	0.45 (0.10–1.90)	0.33 (0.07–1.49)
Bad		0.24 (0.05–0.99)	0.30 (0.07–1.27)	0.17 (0.02–1.26)	0.11* (0.01–0.35)
Very bad		0.03** (0.01–0.36)	0.04* (0.00–0.48)	0.02* (0.01–0.52)	0.01* (0.01–0.35)
**Childhood vaccination(s)**					
No		Ref.	Ref.	Ref.	Ref.
Yes		1.39 (0.62–3.09)	1.37 (0.61–3.09)	1.31 (0.43–3.98)	1.22 (0.39–3.84)
**Trust in health authorities**			1.43* (1.02–2.01)	0.76 (0.43–1.33)	0.67 (0.37–1.21)
**Health behavior models**					
*HBM*					
Perceived susceptibility				1.85** (1.21–2.82)	1.75* (1.12–2.75)
Perceived severity				0.97 (0.49–1.90)	1.01 (0.49–2.07)
Perceived benefits				3.06*** (1.8–5.20)	3.28*** (1.17–6.00)
Perceived barriers				1.32 (0.72–2.41)	1.39 (0.73–2.64)
Cues to action				1.72* (1.09–2.70)	1.55 (0.97–2.47)
***TPB***					
Attitude					1.52 (0.82–2.80)
Subjective norms					1.26 (0.72–2.21)
Self-efficacy					1.55* (1.02–2.37)
**Model Statistics**					
Cox and Snell pseudo *R*^2^	0.16	0.21	0.26	0.40	0.41
Nagelkerke pseudo *R*^*2*^	0.26	0.35	0.36	0.66	0.68

Notes: Each variable with *p <* .*05* in univariate analysis was included in the hierarchical logistic regression model, significant coefficients are shown in bold

*p < .05

**p < .01

***p < .001

### 3.4. Reasons behind unwillingness or undecided to vaccinate

**Fig 2:**
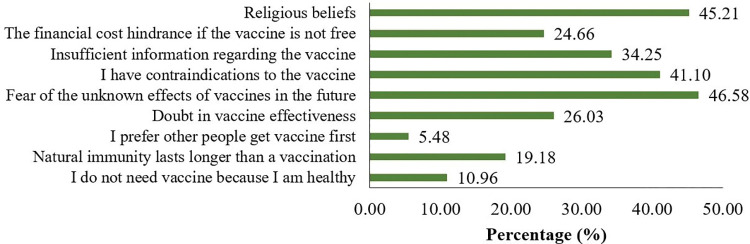
Frequency of possible reasons for COVID-19 vaccine hesitancy (N = 73).

Overall, 18% (N = 73) of respondents refused or were unsure about receiving COVID-19 vaccination. Among them, fear of unknown future consequences (46.58%), religious beliefs (45.21%), and vaccine contraindications (allergy, high blood pressure, diabetes) (41.10%) were the top three reasons for vaccine hesitancy (Fig 2). Around one-fourth of the respondents denied it because of the lack of sufficient information (34.25%), doubt of the effectiveness of vaccination (26.03%), cost of vaccination (24.66%), and firmer belief in the natural immunity system (19.18%) (Fig 2).

## 4. Discussion

### 4.1. Summary of the main findings

Public concern regarding the safety of vaccination and the need for immunization have been raised for as long as vaccinations themselves [53]. However, COVID-19 vaccination reluctance has become a worldwide concern, and this problem will continue to pose a substantial risk to COVID-19 vaccine coverage in the days ahead [54]. In such a worldwide context, socially disadvantaged individuals, mainly urban slum dwellers, are in a precarious position to get the vaccine. Given the high susceptibility to infection due to their poor living condition, the slum people should be given priority for the COVID-19 vaccination [55]. So far, this was the first study to analyze the COVID-19 vaccination acceptance rate, its factors, and reasons for hesitancy among socially disadvantaged people in Bangladesh.

Our study found that more than two-thirds of urban slum respondents intended to get the COVID-19 vaccine. Our finding accorded with a previous study conducted in India, where nearly 65% of urban disadvantaged people were willing to accept the COVID-19 vaccine [55]. In another cross-sectional study conducted among general people of India found that only 27.2% demonstrated vaccine hesitancy, where 35% of the respondents belonged to urban slum areas [56]. Similarly, 66.0% of respondents showed a willingness to get vaccinated in a survey carried out in a slum community of Brazil [57]. On the contrary, a rapid meta-analysis study based on 36 studies conducted in the low and lower-middle-income countries reported the COVID-19 vaccine acceptance rate among the adult population was 58.5% which was a bit lower than our study findings [58]. However, a multi-wave survey conducted continuously for two-week found, 79% positive response to the vaccination among the Bangladeshi adult which was closer to our findings [59]. Fear of contracting COVID-19 may be one of the potential reasons for the high acceptance rate of vaccination. In addition, our survey was conducted around the period when vaccination had already started among other population groups of the country [15]; hence, people were already aware of the benefits of vaccination.

Our study confirmed that individuals with secondary level education were more inclined to receive a vaccination than those with lower education levels or no education. This finding was in line with a previous research [60]. In a separate study, vaccine acceptance in Bangladesh was significantly lower (58.7%) among respondents with no formal education than those with at least one year of schooling [43]. In developed countries like the United States, Poland, and Italy, education along with high income were also considered a positive and substantial predictor of reducing hesitancy among the people [61]. With knowledge on health literacy, educated persons are much more aware of vaccine benefits and have easy access to reliable information which might make individuals more willing to accept the vaccine and likely to trust medical professionals [62].

In our study, we observed that individuals who perceived their health condition as poor were less inclined to accept the COVID-19 vaccine. This aligns with a prior research conducted in South Korea, which demonstrated that individuals with deteriorating health status exhibited 1.38 times higher hesitancy toward the COVID-19 vaccine compared to those in better health [63]. This finding suggests that concerns about potential vaccine side effects may contribute to their hesitancy, as they worry that receiving the vaccine could worsen their health condition [64].

According to the results of our HBM model, vaccine acceptance was significantly influenced by the three factors: perceived vulnerability, perceived benefits, and cues to action. These findings were congruent with the previous studies in Bangladesh and elsewhere [15, 65, 66]. The positive association found between perceived COVID-19 susceptibility and vaccination acceptance indicate that individuals who perceived themselves to be at a higher risk of contracting COVID-19 were more likely to accept the vaccine. This suggests that their understanding of the potential threat posed by COVID-19 motivates them to view vaccination as an essential strategy for self-protection. By recognizing their vulnerability to the virus, these individuals prioritize the benefits of vaccination in reducing their chances of infection and its associated risks. A research in India found that slum dwellers who did not consider COVID-19 a serious threat were less likely to get vaccinated [55]. Similarly, a study conducted in Salvador indicated that a greater perceived risk of COVID-19 reduced vaccine hesitancy [67].

Perceived benefit was also positively correlated to vaccine acceptance in our study. Similar finding was observed in Bangladesh [68] and elsewhere [22, 69]. Another study found personal benefits of immunization had a stronger impact on reducing vaccine hesitancy during COVID-19 [67]. This could be explained by an experiential study that demonstrated selfish and altruistic motivations were highly effective in motivating people to get vaccinated [70]. Recognizing the potential advantages of vaccination, such as minimizing the risk of severe illness, hospitalization, and virus transmission, can strongly influence and diminish vaccine hesitancy [71]. By highlighting the personal benefits of immunization, individuals are more likely to perceive vaccination as a valuable tool in safeguarding their own health and well-being. This emphasis on personal benefits can effectively address concerns or doubts people may have about the vaccine and contribute to increased acceptance rates during the COVID-19 pandemic.

Our study supports previous research indicating that cues to action, such as recommendations or campaigns from doctors, public figures, or government health authorities, had a positive influence on vaccine acceptance decisions [24]. In particular, influential individuals like healthcare personnel play a crucial role in motivating people to get vaccinated [72]. Studies have shown that receiving COVID-19 vaccine information from medical personnel was associated with higher levels of self-efficacy compared to receiving information from coworkers or colleagues [73]. This highlights the importance of healthcare professionals in providing accurate and trusted information to enhance individuals’ confidence and belief in the efficacy of vaccines. While perceived barrier is an important dimension of the HBM model that provides crucial context in shaping vaccination decisions [74], our study did not find perceived barriers to be a significant predictor. One possible explanation could be that individuals in the slum areas might have developed trust in their natural immunity and perceived it as sufficient protection against the virus, leading to reduced perceived barriers to vaccination. A study conducted by the International Centre for Diarrhoeal Disease Research, Bangladesh (icddr,b) found that a substantial portion of the slum population (72%) had already developed coronavirus antibodies [75]. The presence of a relatively high level of natural immunity in the slum population could create a sense of protection against the virus, resulting in reduced concerns about contracting COVID-19. Consequently, this perception of natural immunity might lead individuals to perceive vaccination as less necessary, thus reducing the perceived barriers to getting vaccinated.

In terms of the TPB model, the results also indicate that an individual’s self-efficacy, critical attitudes and behavioral aspects were strong predictor of their willingness to be vaccinated. This result was consistent with a previous study conducted among the general Israeli population who showed higher health literacy by regularly practicing proper public health measures such as frequent hand washing, social distancing, etc. [24]. Indeed, people with good health literacy are more likely to receive the vaccine [62]. Hence the government should emphasize more on public health education programs in the urban slum to educate socially vulnerable individuals on public health measures. However, some other important dimensions like attitudes and subjective norms were not significant in our study. Our study was conducted before the vaccination program started, and slum residents might not have been well-informed about the COVID-19 vaccines. Without sufficient awareness and knowledge about the benefits and safety of vaccination, attitudes towards vaccination might not strongly influence their vaccination behavior. Additionally, as the vaccination program had not yet started at the time of this study, the influence of subjective norms, which is based on perceptions of what others are doing, might not have been as prominent due to the limited exposure to vaccination-related behavior within the community.

Regarding the reasons for vaccine reluctance, respondents cited fear of unknown side effects, religious views, contraindications to vaccination, and insufficient information on the vaccine as the primary reasons for being hesitant toward vaccination. A study conducted in India reported that 30% of participants in Mumbai and 14.8% in Delhi slums people expressed concern about being vaccinated against COVID-19 [76]. Another study conducted among general population of Bangladesh reported a high proportion of people cited the potential side effect of vaccine as a reason for vaccine refusal [77]. Secondly, a strong religious belief was found among the respondents in our study who showed unwillingness to be vaccinated. Some people mistakenly believe that the vaccine contains ’haram’ (religiously forbidden) ingredients like pig-derived products that influence their decision on vaccine acceptance [78]. A similar misconception has been observed previously during ’Polio’ vaccination program [79]. There is also a traditional healing way in African churches, where they pushed their faith healing above medical science by stating that God will heal everyone [80]. Third of all, a significant proportion of respondents reported having health contraindications was one of the reasons for being hesitant toward vaccination. This might be due to the false information in slum settlements regarding the vaccine’s safety and negative effects. Therefore, they were apprehensive that if they received the vaccination in such a medical situation, their health might further deteriorate. A similar study in Indonesia reported that individuals having pre-existing medical conditions showed more hesitancy toward COVID-19 vaccination [81]. Apart from these factors, several controversies and incorrect information have also affected vaccination faith, not only in our current study (34.25%) but also reported in a previous study [53]. Similarly, the slum dwellers of Mumbai had the highest percentage of those who said they didn’t believe in vaccinations (20%), followed by Delhi (7.4%) and Kolkata (6%). In Mumbai, 22% of residents were skeptical of the vaccine’s effectiveness in preventing the epidemic and did not trust the government system [76]. In most of the cases, information concerning vaccinations is frequently not effectively shared, leading to uncertainty and worry about newer vaccines being introduced into the community. This demonstrates that participants are making judgments depending on their comprehension of the facts, and this misinformation might substantially hinder vaccination campaign efforts [82]. In addition, 24.66% of respondents in our study cited vaccination cost as a major factor in refusing to be vaccinated. Respondents from most of the slum settlements in LMICs showed a similar pattern of behavior [57, 76]. Last of all, a widespread misperception was found among the respondents that if a person recovers from a COVID-19 infection, they will obtain immunity, which may contribute to their reluctance to take the COVID-19 vaccine [43].

### 4.2. Strengths and limitations

Our research has some notable strengths. This study’s most vital point is its originality—the combined use of the Health Behavior Model and Theory of Planned Behavior to investigate the determinants of COVID-19 immunization among urban slum residents. These two models provide essential decision-making insights by evaluating what motivates and discourages health-related behavior [24]. Further, most studies on vaccine acceptance issues in Bangladesh were conducted online due to the government-imposed lockdown. This naturally excluded the socioeconomically marginalized population groups who lack the knowledge and access to the necessary device to participate in an online survey. Therefore, the information we gathered from the urban slum-dwellers through a face-to-face interview on this critically important public health issue would be of tremendous use to academics and government authorities. However, some limitations should be acknowledged before interpretation. First, the results of this research provide a snapshot of the situation before the rollout of vaccinations among urban slum dwellers in Bangladesh. However, people’s intentions may vary since their disease risk perception fluctuates over time. Second, we could not include other socioeconomically disadvantaged groups with similar healthcare access disparities. Third, the cross-sectional nature of the research prevented us from establishing cause-and-effect relationships between the variables of interest. However, future studies should focus on a longitudinal study on this particular issue, which may help researchers determine how health-related policies affect these factors. Further, our sample is predominantly female since most participants were homemakers when data was collected. Finally, we studied only two big cities of the country, including the capital, but not all the slums. Thus, the lack of a nationwide sample may limit the generalizability of the results.

## 5. Conclusion

This study found that two-thirds of slum dwellers were willing to accept COVID-19 vaccination. Further, our research found that vaccination rates vary by education level, perceived health condition, perceived susceptibility, perceived benefits, cues to action, and self-efficacy. Fear of unknown side effects, religious views, vaccination contraindications, and inadequate vaccine information were the main reasons for vaccine hesitancy. These findings could help policymakers to design appropriate measures to increase vaccination acceptability among the socially disadvantaged people in the urban area of Bangladesh. Specifically, individuals with poor health conditions should be urged to get the COVID-19 vaccination. In addition, the government and policymakers of Bangladesh should start a public health education campaign for slum dwellers that emphasizes the perception of vaccine benefits and the severity of the disease. Furthermore, the reduction in vaccination may reverse through an effective communication strategy to build community confidence, increase understanding, and debunk misconceptions about the safety and effectiveness of vaccinations. Hence, it is imperative for the Ministry of Health to allocate additional resources towards comprehensive public awareness campaigns, prioritizing inclusivity in vaccination efforts, and fostering a culture of sharing positive experiences with the COVID-19 vaccine. In this context, the active engagement of influential figures, including healthcare professionals, politicians, celebrities, as well as community members, religious leaders, and young individuals, becomes paramount. Their voices and testimonials can serve as powerful catalysts to highlight the significance of vaccination and inspire individuals to embrace the COVID-19 vaccine.

## Supporting information

S1 Data (XLSX)
